# Screen language dominance: “my memory is completely English, but I speak Swedish”—a case study

**DOI:** 10.3389/fpsyg.2026.1886528

**Published:** 2026-10-13

**Authors:** Emma Forsblom, Viveka Lyberg Åhlander, Stina Sundstedt

**Affiliations:** 1 Speech and Language Pathology, Department of Humanities, Faculty of Human and Social Sciences, Åbo Akademi University, Turku, Finland; 2 Logopedics, Phoniatrics and Audiology, Department of Clinical Sciences, Lund University, Lund, Sweden

**Keywords:** autism spectrum disorder, echolalia, language acquisition, screen accent, screen language dominance, unexpected bilingualism

## Abstract

**Introduction:**

Children with autism spectrum disorder (ASD) may go through phases where they reuse utterances from screens, adopt an accent acquired from screens, or develop language skills in languages not used in their environment.

**Methods:**

This case study explores *screen accent* and *screen language acquisition* through “Stella”, a nine-year-old Swedish-speaking girl with ASD who acquired English through screens. Data were collected through language assessments (CELF-5) that were conducted by speech-language pathologists, as well as through a parent interview, medical records, and home videos.

**Results:**

Stella used only English from age three to five, only Swedish from age five to nine, and at age nine she mainly uses Swedish, with English in specific contexts, like online gaming. The findings indicate that Stella is showing *screen language dominance*, as recent assessment results and her early developmental history indicate a greater proficiency in the screen language (English) compared to the language spoken in her environment (Swedish). This is noteworthy considering the range of support Stella has received to develop her Swedish skills.

**Discussion:**

This study contributes to future research by describing and defining terms related to screen language acquisition and by addressing the potential negative impact of screen language dominance on communicative participation. At the same time, although screens are often associated with negative outcomes, findings from the present case study and recent research indicate that some children with ASD may effectively acquire language through screens, indicating potential benefits for language development. Taken together, these findings underscore the need for further research including children with and without ASD.

## 1 Introduction

Based on anecdotal observations, it appears that some young children with autism spectrum disorder (ASD) as well as children with typical development learn a language, like English, through screens. This has also been reported in both Finnish and Swedish newspapers, where health professionals and educators are concerned about how children in Finland and Sweden have increasingly started to use English that they have learned through YouTube (Mustonen, 2024; Tenfält, 2024). According to a study by Larsson et al. (2023), some children in Swedish preschools use English, most likely learned through screens, when communicating. Although typically developing children in Finland and Sweden seem to acquire some English through screens as a second language, anecdotal observations by Finnish and Swedish speech-language pathologists (SLPs) as well as some studies (Vulchanova et al., 2012; Kadiri and Anasse, 2023; Zhukova et al., 2023; Dumont et al., 2024, 2025; Hindi and Meir, 2025) associate atypical use or screen-based acquisition of a foreign language with autistic traits. Through a careful examination of a child’s language development and the terminology used in previous research, this case study seeks to expand and deepen the understanding of screen-based language acquisition in ASD.

## 2 Background

ASD is a neurodevelopmental condition associated with atypical social communication, intense interests, and restricted or stereotypical behavior (American Psychiatric Association, 2013). Furthermore, ASD is characterized by discrepancies in language and cognitive profiles (Nevill et al., 2019), as well as atypical prosody (Patel et al., 2020). Language acquisition in children with ASD appears to deviate from the way typically developing children acquire languages, for example in relation to joint attention (Kissine et al., 2023) and echolalia (Luyster et al., 2022). Echolalia, which is instant or delayed repetition of utterances heard in everyday life or on screens, occurs frequently in children with ASD (Luyster et al., 2022). It has been suggested that, at the beginning of language development, children with ASD may reuse utterances in their original form but gradually start modifying the utterances to make the response more appropriate to the situation (Mottron et al., 2021). Previous research suggests that modifications of repeated utterances, including lexical and grammatical changes, may reflect developmental progress (Cohn et al., 2022). Given the language and communication differences associated with ASD, the role of multilingualism in language development has received increasing research attention. However, current evidence does not support the assumption that bilingualism negatively affects the language and communication skills of children with ASD (Meir and Novogrodsky, 2020; Gilhuber et al., 2023). Instead, bilingualism appears to affect children with ASD and typically developing children similarly (Meir and Novogrodsky, 2020; Gilhuber et al., 2023).

In addition to multilingualism, screen exposure has been discussed as a factor that may influence language development. Children with ASD have been found to have higher levels of screen exposure compared to children without ASD (Dong et al., 2021; Liu et al., 2025). According to Dong et al. (2021), typically developing children at around 3 years of age have, on average, less than 1 h of screen time per day, whereas children with ASD of the same age have, on average, around 3 h of screen time per day. Some studies suggest that high levels of screen exposure are associated with deficits in social skills and autistic-like behavior, but the direction of causality in this association has not yet been determined (Alrahili et al., 2021; Chen et al., 2021). Hill et al. (2020) indicate that there seems to be an association between screen exposure and decreased language development in children with ASD.

Beyond children with ASD, little verbal interaction during screen exposure combined with early introduction to screens and high screen exposure in the first 2 years of life appears to be associated with diminished language development in typically developing children (Supanitayanon et al., 2020). Previous studies argue that typically developing children acquire more vocabulary through interactions with adults than through screen exposure (Krcmar et al., 2007). According to Krcmar et al. (2007), children may need some language comprehension skills before being able to understand television programs and to maintain attention. Furthermore, while vivid programs might be entertaining for children, they may not support language development as children can struggle to know where to direct their attention (Krcmar et al., 2007). Sundqvist et al. (2025) also consider that screen use has a negative association with vocabulary and grammar skills, and that watching non-native language content negatively impacts native language development.

### 2.1 Screen-based language acquisition in children with ASD

Notwithstanding findings suggesting that screen exposure is associated with decreased language development in children’s mother tongue, some studies have reported second language acquisition from screens in children with ASD. Currently, there are three case studies about screen-based language acquisition in which all participating children have ASD (Vulchanova et al., 2012; Kadiri and Anasse, 2023; Zhukova et al., 2023), as well as three group studies in which no typically developing children were observed to have acquired a foreign language through screens; instead, this was observed only in the ASD group (Dumont et al., 2024, 2025; Hindi and Meir, 2025). However, a recent study observed screen-based acquisition of letters and numbers even among typically developing children, although the prevalence was significantly higher among children with ASD (Gagnon et al., 2026).

Some studies refer to this phenomenon, where children acquire a foreign language through screens, as *unexpected bilingualism* (Zhukova et al., 2023; Dumont et al., 2024, 2025; Ostrolenk et al., 2025; Gagnon et al., 2026; Mottron et al., 2026). The term unexpected bilingualism, however, lacks a clear and consistent definition, and this complicates the use of the term and comparison between studies. The term is composed of two elements, *unexpected* and *bilingualism*, each of which allows for considerable interpretive variation. It can be questioned what features are considered to be unexpected (e.g., non-interactive language learning or atypical use of the language) in these children’s language acquisition. The use of the term bilingual is also problematic, as some of these children seem to speak and understand only the foreign language acquired through screens, while bilingualism would presuppose that the child has knowledge of two different languages. Individual studies have also referred to the phenomenon as *spontaneous foreign language acquisition* (Zhukova et al., 2023) and *non-interactive language acquisition* (Hindi and Meir, 2025). While these terms highlight certain aspects of the phenomenon, neither explicitly identifies screen exposure as the primary source of language input.

To address these limitations and the inconsistent use of terminology, this study introduces a set of novel terms, in which a language only heard through screens and not spoken in the child’s environment is referred to as *screen language,* and the process of learning a language predominantly from screen input is referred to as *screen language acquisition.* Our suggestion is that a screen language is classified as such when it is acquired predominantly through non-interactive screen exposure rather than through regular interpersonal interaction with caregivers, family members, peers, or educational staff. Thus, a language remains a screen language even if interpersonal communication in that language develops later, provided that screen exposure was the primary source of acquisition. Having more developed language skills in the screen language compared to the language spoken in the child’s environment is referred to as *screen language dominance*. We define screen language dominance as a higher level of proficiency in the screen language than in the environmental language, demonstrated by a clinically meaningful difference as evidenced by assessment results, language use, or parental report. The presence of screen language dominance may vary over time as the child’s language skills develop.

In addition to foreign languages, children with ASD may also have a remarkable mastery of an accent or a linguistic variety acquired through language input on screens, e.g., a child uses a linguistic variety featured in television programs instead of the linguistic variety spoken in the environment (Kissine et al., 2019; Abd El-Raziq et al., 2024; Francis et al., 2024; Beccaria et al., 2025). To our knowledge, no established term for this atypical accent or linguistic variety exists in the literature, and therefore, we refer to this as *screen accent*. Screen accent may include phonological, prosodic, and dialectal features acquired primarily through screen exposure. To qualify as screen accent, these features should be present as a noticeable and recurring aspect of the child’s speech rather than as isolated instances of imitation. Together, these terms provide a coherent framework for describing language acquired through screens, from the source of input to potential linguistic outcomes.

The existing studies have proposed varying explanations for screen language acquisition and screen accent. Pragmatic difficulties frequently occur in children with ASD, and therefore they may struggle to understand that they use a different linguistic variety than other children (Abd El-Raziq et al., 2024). For children with ASD, verbal interaction is challenging, and delayed echolalia may provide a non-interactional means of detecting phonological patterns from repeated content (Kissine, 2021). In the same vein, it has been proposed that children with ASD often show a detail-focused processing style, reflecting a bias toward local rather than global processing, which may facilitate attention to recurring linguistic structures in screen-based language input (Vulchanova et al., 2012). According to Francis et al. (2024), screen accent is not due to a greater exposure to the linguistic variety nor to any family-related factors. However, the repeated language content and lack of social factors may facilitate analysis of the structure of the language and thereby facilitate learning the language (Dumont et al., 2024; Francis et al., 2024; Gagnon et al., 2026). It has been suggested that some children with ASD may rely less on social interaction in language learning and instead be particularly attentive to perceptual regularities in language input, including screen-based language input (Mottron et al., 2026). This account proposes that sensitivity to recurring perceptual structures may facilitate the detection of phonological, lexical, and grammatical patterns in highly repetitive and structured input. Furthermore, previous studies suggest that the children’s special interest may contribute to language acquisition (Vulchanova et al., 2012; Kissine et al., 2019; Zhukova et al., 2023; Hindi and Meir, 2025). Likewise, children with ASD have themselves described that screen language acquisition is automatic, as they have been motivated to know more about their special interest (Hindi and Meir, 2025). It has also been proposed that screen language acquisition may involve a self-reinforcing process, whereby increasing proficiency in a language motivates children to seek further exposure to that language (Gagnon et al., 2026). The children’s parents have even considered an emotional event to be the triggering factor for the language switch to a screen language (Hindi and Meir, 2025). Beyond possible explanations, it is not yet known whether this phenomenon is restricted to second language acquisition (Kissine et al., 2023), nor is it known whether acquiring a foreign language could act as a bridge to developing proficiency in the mother tongue (Hindi and Meir, 2025).

### 2.2 Knowledge gap and the current study

Despite an increasing number of studies, the relationship between language input through screens and language development in children with ASD is still unclear. Studies describe children who have acquired a screen language (a foreign language acquired predominantly through screens), suggesting that for at least some children, screens may after all facilitate language development (Vulchanova et al., 2012; Kadiri and Anasse, 2023; Zhukova et al., 2023; Dumont et al., 2024, 2025; Hindi and Meir, 2025). While studies indicate that children may acquire a foreign language through screens, there is no consensus on how to explain this type of language acquisition or screen language dominance. Furthermore, there are no clear clinical guidelines to support health professionals in providing evidence-based advice to caregivers of children with ASD regarding screen language input and screen exposure. Currently, there is a lack of clear terminology distinguishing between screen language acquisition and screen language dominance. This distinction is essential in both research and clinical practice when differentiating children with screen language dominance, who may be at risk of reduced communicative participation, from children who acquire language through screens while maintaining sufficient skills in the environmental language.

The present study forms the first part of a larger research project focusing on screen language acquisition and aims to provide insight into screen language acquisition with a case of a girl with ASD. The case describes Stella (pseudonym), living in a Swedish-speaking family in Finland, who showed screen language acquisition, screen language dominance, and later a screen accent. To our knowledge, no case from the Nordic countries or a Swedish-speaking environment has previously been described in the literature. Stella’s switch from speaking only a foreign language acquired through screens to using only the language spoken in the environment appears to represent a previously undocumented developmental trajectory. The aim is to expand understanding of screen language acquisition by describing both the factors indicating screen language dominance and Stella’s language switch, and by assessing Stella’s proficiency in the screen language and the environmental language. Furthermore, this study aims to problematize and contribute to clarifying the terminology concerning screen language acquisition.

This study aimed to answer the following research questions:

1. How was screen language dominance manifested in Stella’s language use, and at what developmental stages?
2. What levels of proficiency in Swedish and English did Stella show according to standardized testing at age nine?

## 3 Materials and methods

### 3.1 Participant

Stella is a nine-year-old girl from Finland. She is the youngest child in a Swedish-speaking family, and she has three older siblings. Swedish is an official minority language in Finland. Stella grew up in a predominantly Swedish-speaking rural area, and Finnish was not used in her family, educational setting, or daily social environment. Her only exposure to Finnish was through Finnish language lessons in school, typically two lessons per week. As English is introduced as a foreign language only after fourth grade, Stella had not received any formal instruction in English at the time of testing. Even though Stella has grown up in a monolingual Swedish-speaking environment, English appears to have unexpectedly been her dominant language from a young age. Stella received a diagnosis of atypical ASD when she was 5 years old.

### 3.2 Procedure, conceptualization and analysis

The data collection in this study consists of a parent interview, medical records, home videos, and assessments of language skills and social responsiveness. Ethical approval was received separately for the parent interview and medical records, and the home videos and assessments, from the Board for Research Ethics at Åbo Akademi University. The parents provided written informed consent before the data collection began, and Stella gave verbal consent to participate in the study. The parents received a detailed data protection notice, which included information about their right to discontinue participation at any time.

The parent interview was conducted with the mother in a video meeting by the first author at the beginning of 2024. The interview lasted 3 h and was transcribed after the session. The interview was supplemented by additional questions asked in conjunction with one of the test sessions in 2025.

The mother provided the researchers with Stella’s medical records from ages 2:5 to 8:2 (years: months). These records contain data from the first meeting with the pediatrician when Stella was 2:5 years old, all the notes from the speech and language therapy assessments conducted from age 2:7 to 8:2 and notes from the SLP and occupational therapist who provided weekly therapy, written at the end of therapy periods. In addition, they include the pediatric neurologist’s notes from age 4:1 and all health professionals’ notes from the team assessment from age 5:4, namely the aforementioned pediatric neurologist, a psychologist, an occupational therapist, and an SLP who had not assessed Stella on other occasions. Consequently, the medical records contain information from three different SLPs who have described Stella’s speech and language development independently of each other. The medical records were pseudonymized before storage. Language use and level in the medical records are presented based on health professionals’ assessments and descriptions, which were reviewed and summarized by the first author.

The home videos were obtained from the parents in 2025 to illustrate Stella’s language development over the years. The mother gave access to a total of 17 home videos, totaling approximately 15 min, from ages 1:0 to 9:7. It should be noted that only a limited number of home videos from Stella’s early years were available, constraining selection to the recordings that had been preserved. All videos provided were included in the analysis, and the videos were transcribed orthographically. The language level in the home videos was categorized according to the structural complexity of the child’s verbal production, ranging from babble to fluent connected speech. The categories were selected to represent clearly distinguishable levels of verbal production and were considered sufficiently distinct to allow reliable classification. In addition, we analyzed the language used by Stella in the video recordings. Two independent raters, both master’s students in speech and language pathology, reviewed all 17 video recordings and classified the language used and the language level based on these predefined coding categories. Cohen’s kappa was used to assess inter-rater reliability between the two independent raters, taking into account agreement occurring by chance (Cohen, 1960). Disagreements were subsequently discussed by the two raters and the first author to reach a consensus. During these discussions, it became apparent that the coding guidelines needed further clarification regarding whether language level should reflect the child’s highest observed level or the level most frequently demonstrated within a video. A consensus decision was made to code language level according to the highest observed level, and all videos were subsequently reviewed to ensure consistency with this criterion. In addition, singing attempts were coded as singing. In one video, language level was coded as not applicable because the reduced intelligibility and background noise made reliable classification difficult.

To address the research questions, we chose to examine screen language dominance using formal assessments, which became feasible once Stella was able to cooperate with formal testing procedures. Screen language dominance was operationalized as a higher proficiency in the screen language than in the environmental language, based on measures of both language comprehension and production. A difference of at least one standard deviation was considered indicative of meaningful dominance. Standardized language assessments were conducted in person with Stella in both Swedish and English by two certified and experienced SLPs using the Swedish and English versions of Clinical Evaluation of Language Fundamentals, Fifth Edition (CELF-5; Wiig et al., 2013). The test was first administered in Swedish when Stella was 9:5 and in English at age 9:8, and the same subtests were administered in both languages. The three-month gap was considered sufficient to minimize carryover. The selected subtests were chosen to provide a broad evaluation of Stella’s language abilities across both languages. To limit the duration of the assessment, subtests included in the Language Content Index were not administered. The same subtests were used in Swedish and English to assess comparable language domains and facilitate cross-linguistic interpretation of performance. Normative bands were used to describe Stella’s performance relative to the normative sample of each language version. They were not intended to support direct comparisons between the Swedish and English editions. The SLP who conducted the test in English has a high level of proficiency in English. In addition, the scores from the test were reviewed by one of the authors. To get an updated description of Stella’s autistic traits and social skills at age 9:5, the mother completed the Social Responsiveness Scale, Second Edition (Constantino and Gruber, 2012). This information was considered relevant as previous studies have suggested that characteristics associated with ASD may be related to screen language acquisition. In addition, several years had passed since Stella received her ASD diagnosis. The test sessions were video recorded, which made it possible to analyze spontaneous speech in both languages and Stella’s own thoughts about speaking English. The video recordings from the test sessions (about 5 h in total) were transcribed orthographically. Findings were triangulated across assessment data, parent interview, medical records, and home videos to strengthen the credibility of interpretations regarding language development and language dominance.

### 3.3 Developmental history

The developmental history presented below is based primarily on the parent interview and supplemented by information from the medical records. Stella’s mother had elevated liver values at the end of pregnancy, but no other abnormalities were noted during pregnancy or delivery, and Stella was born healthy. Stella was a highly demanding baby and the mother noticed early that Stella behaved differently from her older siblings. Among these atypical features, the mother noted that Stella slept poorly, had atypical eye contact, was afraid of adults, and was remarkably sensitive to sounds. Furthermore, Stella reached the motor developmental milestones slightly later than her siblings, but no major deviations were noted.

Stella was often uneasy as a baby, and thus the parents perceived that playing music on YouTube would calm her down. Consequently, Stella began to listen to music frequently, at first in Swedish, and after she discovered how to change the song on her mother’s smartphone herself, she preferred English songs. *Twinkle, Twinkle, Little Star* was her favorite song as a baby. In the interview, the mother explained that she thinks that the English language she heard on screens provided comfort for Stella, whereas the Swedish environment was perceived as a bit dangerous. Stella was a fearful and guarded child, but when she started daycare at age 1:8 her behavior changed and she became very active. Hence, letting Stella watch videos on a screen was the only way to manage daily life. The mother estimated that Stella’s daily screen time had been 2 to 3 h ever since early childhood.

The mother raised concerns about Stella’s development several times with healthcare professionals and relatives. At age 2:6, Stella received a referral for speech and language therapy and occupational therapy assessments. At age four, Stella visited a psychologist and soon after that she visited a pediatric neurologist, who gave her the diagnosis “challenges in development, note autistic symptoms”. A multidisciplinary assessment was done when Stella was at age 5:4 and then she received the diagnosis “atypical autism spectrum disorder”. The team considered her diagnosis “atypical” due to lack of extensive restricted, repetitive, and stereotyped behavior patterns.

### 3.4 Description of language development

Below is a description of Stella’s language development and how the use of English (screen language) and Swedish (environmental language) has varied over the years, based on the parent interview, medical records, and home videos. For an overview, see Figure 1.

**Figure 1:**
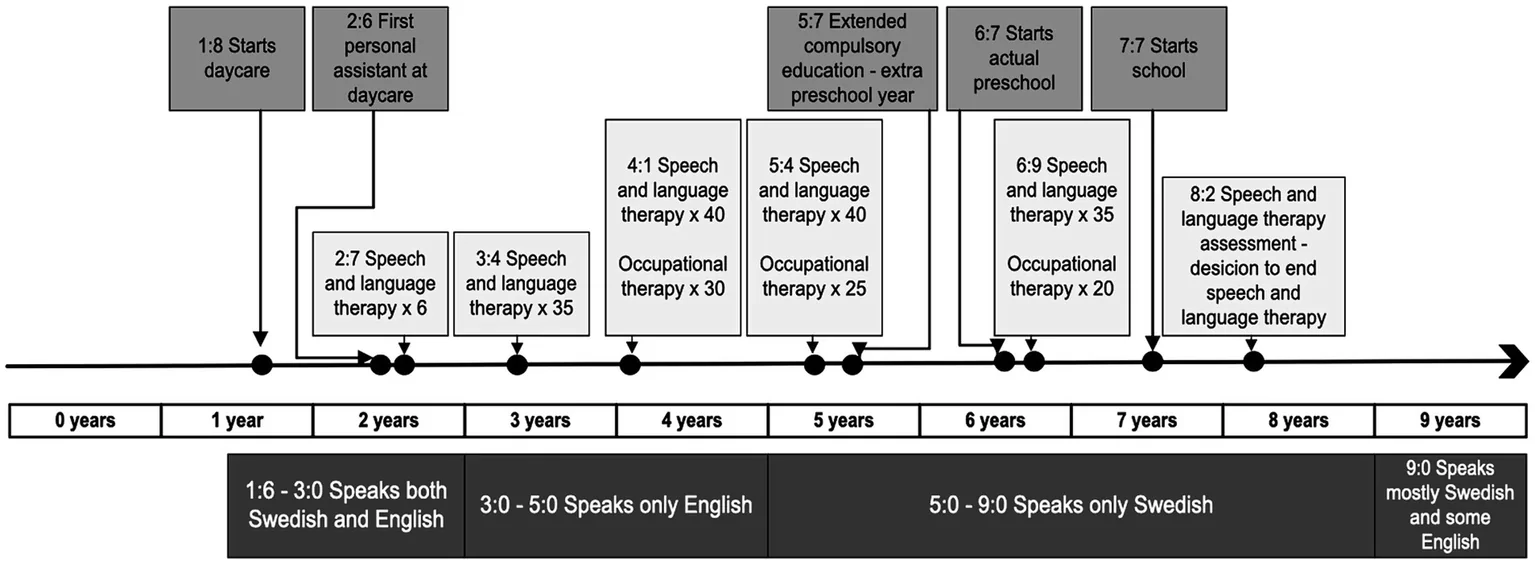
Timeline of important milestones in Stella’s development. The darkest gray boxes show which language Stella spoke at the time, the lightest gray boxes show how many 45–60-min therapy sessions Stella received per year at different ages, and the medium gray boxes give information about daycare and school.

According to the mother, at the beginning of language development Stella was using both Swedish and English. Stella was not speaking much, but she was singing and communicating through songs. When she was singing, some words were clear, but the rest were nonsense words with English phonology. At age 2:6, Stella used English almost exclusively. At the time, she started watching the children’s program “Peppa Pig,” and she began to imitate the character Peppa’s voice. According to the mother, Stella’s speech contained many words and phrases from “Peppa Pig”. Nevertheless, these utterances were not always instances of direct echolalia, as Stella often modified them to fit the communicative context. Because it seemed that Stella understood English better, the mother occasionally clarified in English, and the older siblings could sometimes speak some English to Stella because they found it easier. This was the only input Stella received in English from her environment. It was always on Stella’s initiative, and the interpersonal exposure began only after acquisition. According to the mother, Stella was speaking fluent English when she was 4–5 years old, and her language skills were continually developing. In the medical records, the pediatric neurologist wrote that when Stella was 4:1, she had an atypical development and considered the consistent use of English to be striking.

Both the mother’s interview and the medical records indicate that Stella only spoke Swedish between the ages of five and nine, but Stella continued to watch shows in English. When Stella switched to using Swedish, she started with single words, but quickly began to put words into sentences. After a while, she refused to speak English at all. In the interview, the mother mentioned that at the beginning Stella pronounced Swedish words with an English accent. The pediatric neurologist noted at age 5:4 that Stella had an unusual Swedish accent that was used in Sweden and not in the Swedish-speaking parts of Finland.

When Stella turned 9 years old, she began to use English again in specific contexts, primarily during online gaming with some friends who had also learned English by then. Nevertheless, she did not speak English with her family or at school, and all English still appears to be acquired only through screens, as formalized English lessons are not introduced until fourth or fifth grade in Swedish-speaking schools in Finland. At age 9:5, the mother estimated that Stella’s stronger language was Swedish.

### 3.5 Support for the development of the Swedish language

Over the years, Stella has received a range of support to develop her Swedish skills, including speech and language therapy, occupational therapy, and additional support in daycare and school. Stella started speech and language therapy immediately after the first assessment when she was 2:7, and she saw an SLP 35–40 times a year until she turned eight. The goal of the speech and language therapy was to focus on social interaction and the communicative use of language, as well as supporting language comprehension and expressive language in Swedish. At the assessment when Stella was 8:2 years old, no further speech and language therapy was recommended because the linguistic and pragmatic difficulties no longer had a significant impact on Stella’s everyday life and participation. Stella received a recommendation for occupational therapy after the first assessment at age 2:7, but the start was delayed until Stella was 4:3 years old due to difficulties finding an available therapist. Stella saw an occupational therapist 20–30 times a year until just before she turned eight.

Stella received her first personal assistant at daycare when she was 2:6 years old and continued to have a personal assistant in school. In addition, Stella has seen a special education teacher regularly since daycare. When Stella was diagnosed with ASD, a decision was made to extend her compulsory education, and therefore Stella attended an extra preschool year prior to the regular one. In addition to the extra year of preschool, Stella also participated in a school preparation program with educational tasks provided at home by the mother, for children who need extra support (FHille; Folkhälsan, n.d.).

## 4 Results

### 4.1 Home videos

Below is a systematic description of language use and an analysis of language level observed in the home videos. To evaluate the reliability of the coding, inter-rater agreement was calculated for language use and language level. For the language spoken in the video, the two raters showed 88.2% agreement, with a Cohen’s kappa of *κ* = 0.821, indicating almost perfect agreement (Landis and Koch, 1977). For language level, the two raters showed 64.7% agreement, with a Cohen’s kappa of *κ* = 0.566, indicating moderate agreement (Landis and Koch, 1977). The ratings presented in Tables 1, 2 are based on consensus ratings established following the independent ratings. Language use and level in the home videos from the period when Stella was between 1 and 5 years old are presented in Table 1.

**Table 1:** Language use and level in home videos (ages 1–5).

Age	Language	Language Level	Transcription of Stella’s utterances and context
1:0	Not applicable	Babble	“Va, va, dä, dä…” – Stella’s vocalizations consist only of babble; mother speaks Swedish.
1:10	Not applicable	Song or rhyme	“Jako, so du, so du…” (target: “Broder Jakob, sover du, sover du”) – Music is playing in the background and Stella is singing along. Pronunciation is unclear.
1:11	Swedish and English	Song or rhyme	”Twinkle, twinkle, li-e sta, how wa wa…” – Stella sings short snippets in both Swedish and English. Pronunciation is unclear, but the song and language can be recognized. Grabs a decorative star while singing *Twinkle, Twinkle, Little Star*.
2:6	English	Single words	“Two, three, four…” – Stella counts to ten in English with a rising pitch for each number.
2:10	Not applicable	Not applicable	– Stella’s speech is largely unintelligible, but she has English-like phonology. Father speaks Swedish.
3:4	English	Phrases or short sentences	“Jump, jump!”, “Mommy is trying, come on, come on!” – Stella is jumping on a trampoline and asking her mother to join her. Mother speaks Swedish, but Stella speaks English. These phrases resemble expressions commonly used in “Peppa Pig”; however, they are adapted to fit the communicative context.
3:4	English	Phrases or short sentences	“Now we twinkle” – Stella is in bed, and it seems like she wants to have the telephone. Mother speaks Swedish, but Stella speaks English.
3:11	English	Song or rhyme	“Happy birthday to you…” – Stella sings *Happy birthday* in English.
3:11	English	Song or rhyme	“Happy birthday!”, “Yeah, yeah, yeah.” – Mother asks in Swedish for Stella to sing. Stella speaks English.
4:10	English	Phrases or short sentences	“Thank you, daddy!” – Stella is skiing. Parents speak Swedish, but Stella speaks English.
4:10	Not applicable	Not applicable	– Stella is skiing and humming the melody of *Jingle Bells*.
5:0	English	Phrases or short sentences	“Yes, let us try!” – Stella plays with a toy bathtub and turns on music after the father asks about it. Father speaks Swedish, but Stella speaks English.

**Table 2:** Language use and level in home videos (ages 5–9).

Age	Language	Language Level	Transcription of Stella’s utterances and context
5:1	Swedish	Single words	“Väckarklockan” (the alarm clock), “Stol” (chair), “Telefon” (telephone), “Mattan” (the carpet) – Stella names pictures in Swedish. An English accent can be heard, and some of the words are produced in definite form and some in indefinite form.
5:5	Swedish	Phrases or short sentences	“Jag kan, jag kan, titta!” (I can, I can, look!) – Stella is riding a bicycle. Parents and Stella speak Swedish.
7:0	Swedish	Fluent connected speech	“Å sist har jag några målarböcker.”, “Ett, två, tre, vänta, jag fick två Nalle Puh-böcker, fyra…” (And finally, I have some coloring books. One, two, three, wait, I got two Winnie-the-Pooh -books, four…) – Stella shows objects to her father. Her Swedish skills have developed, but the Swedish screen accent and the influence of English (e.g., “å sist,” ‘and finally’; “vänta,” ‘wait’) can still be heard.
9:7	English and Swedish	Fluent connected speech	“That’s what I feel when you say that you are bad at drawing.”, “You do not have… she does not have to be a monkey if you want.” – Stella talks to her cousin through a video call while playing an online drawing game. At the end of the video, the cousin switches to Swedish, and Stella follows by responding in Swedish.
9:7	English	Fluent connected speech	“What would happen if I said yes?”, “The arms were actually kind of cute.” – From the same discussion with the cousin as above.

Language use and level in the home videos from the period when Stella was between 5 and 9 years old and had started to speak Swedish are presented in Table 2.

### 4.2 Description of language level and previous language assessments based on medical records

A structured overview of health professionals’ descriptions of Stella’s language level between 2:5 and 8:2, based on the medical records, is presented in Table 3.

**Table 3:** Language use and described language level based on medical records.

Age	Professional source	Language use and description
2:5	Pediatrician	English > Swedish – Stella primarily used English during the visit and sang mostly in English. She was able to name words in both Swedish and English.
2:7	SLP	English > Swedish – Expressive vocabulary was reported to be larger in English; Stella could name, for example, colors and animals in English, while only a few words were produced in Swedish.
2:10	SLP	English > Swedish – Stella did not consistently follow directions, which prevented assessment of language comprehension and language dominance. During the session, Stella mainly produced single English words, but according to the mother, Stella was using phrases in English at home. Stella had correct English pronunciation.
3:10	SLP	English > Swedish – In conversation it seemed like Stella’s receptive language had improved. Stella was speaking English in sentences and was using English phonology, even in Swedish names and words. Stella pronounced the names of familiar people as they are pronounced in English, and for names without an English equivalent, she created variants that fit English phonology (e.g., *Jansson* pronounced as *Jansee*). Longer phrases and more advanced grammar were observed in English, which was considered her stronger language at this time. Stella also translated parts of the examiner’s speech into English and reformulated test prompts rather than complying with task demands.
4:1	Pediatric neurologist	English > Swedish – Development was described as atypical, with consistent use of English noted as striking. According to daycare reports, Stella appeared to understand Swedish, but she responded in English.
5:4	Multidisciplinary assessment	Swedish > English – Developmental challenges were noted across domains, especially linguistic, and Stella’s language usage seemed more like commenting than purposeful communication. An unusual Swedish accent was observed. Some words were pronounced with an English accent. The SLP noted incomplete sentence structure (e.g., “Gråter som hunden tar en sko” [“Cries like a dog taking a shoe.”]), grammatical errors with word inflection (e.g., “bilet, ögor, halsduket” [sic; correctly “bilen, ögon, halsduken”]), and contextually inappropriate word use. Swedish language skills were reported to be significantly below age expectations.
6:3	SLP	Swedish > English – Stella was reported to communicate exclusively in Swedish at this time and was described as benefiting from ongoing speech-language therapy.
8:2	SLP	Swedish > English – Residual influence of English was noted in Swedish speech, including frequent use of English discourse markers (e.g., *well*). Some utterances appeared to be direct translations from English.

*“*>” indicates the language used more frequently during the visit and generally preferred by the child at the time, based on descriptions in the medical records, not assessed language proficiency.

A recurring observation in the medical records was difficulty administering standardized tests, which meant that assessments could not be completed in either Swedish or English before age 8:2. Standardized tests were among those attempted at age 3:10 by the SLP and at age 5:4 by various health professionals in the multidisciplinary assessment. All the health professionals’ notes indicated that standardized tests were difficult to perform because Stella insisted on doing things on her own terms and her motivation fluctuated.

The first standardized assessment that was successfully performed was the CELF-4 in Swedish at age 8:2. At that time, Stella’s receptive language was within the lower range of average and her vocabulary and semantics were just below the average range in Swedish, according to the medical records. After some persuasion, Stella agreed to do a vocabulary test in English, but she did not agree to have a conversation in English. When the SLP tested Stella’s vocabulary in English, the result was at the same level as in Swedish, even though Stella had not spoken English to anyone in 3 years. This English vocabulary test is the only measure from a standardized assessment that was administered in English prior to the data collection of the present study.

### 4.3 Language assessment conducted for the present study

The subtests included in the Core Language Score, the Receptive Language Index, the Expressive Language Index, and the Language Memory Index were selected from the CELF-5. As shown in Figure 2, Stella’s Core Language Score and all the index scores were higher in English than in Swedish. The Core Language Score (standard score, SS = 90) and the Receptive Language Index (SS = 88) were at average level for an English-speaking nine-year-old child, and the Expressive Language Index (SS = 85) and Language Memory Index (SS = 83) were classified as borderline in English. In Swedish, Stella’s scores were highest in the Expressive Language Index (SS = 79), which was the only index score that was classified as borderline, whereas the Core Language Score (SS = 74), the Receptive Language Index (SS = 71), and the Language Memory Index (SS = 73) were classified as low.

**Figure 2:**
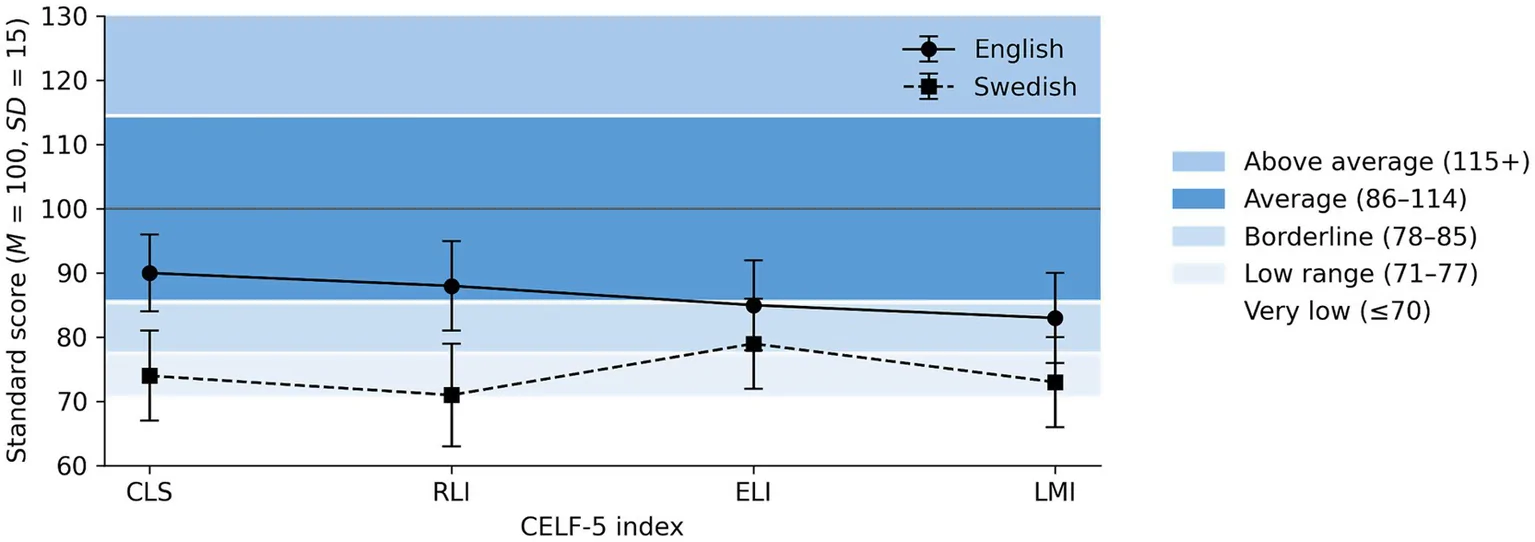
Stella’s results on the Core Language Score and Index Scores on CELF-5 in English (solid line) and Swedish (dotted line). Scores are reported as Standard Scores (mean = 100, SD = 15). The shaded bands represent CELF-5 normative categories. CLS = Core Language Score, RLI = Receptive Language Index, ELI = Expressive Language Index, LMI = Language Memory Index.

Stella’s results on the individual subtests in both languages are shown in Figure 3. Stella’s scores on all other subtests were higher when the subtest was administered in English, except in Sentence Assembly, where the scores were equal (Swedish raw score, RS = 3, scaled score, ScS = 7, 95% confidence interval, CI = 5–9; English RS = 4, ScS = 7, CI = 5–9). In Swedish, the subtests Word Classes (RS = 19, ScS = 7, CI = 4–10), Recalling Sentences (RS = 33, ScS = 7, CI = 5–9), and Sentence Assembly were classified as borderline, whereas the subtests Following Directions (RS = 10, ScS = 5, CI = 3–7), Formulated Sentences (RS = 21, ScS = 6, CI = 4–8), and Semantic Relationships (RS = 1, ScS = 4, CI = 2–6) were classified as low or very low. Hence, all results were below the average level for a nine-year-old whose mother tongue is Swedish. In contrast, in English, the subtests Word Classes (RS = 29, ScS = 12, CI = 10–14), Formulated Sentences (RS = 29, ScS = 8, CI = 6–10), and Recalling Sentences (RS = 40, ScS = 8, CI = 6–10) reached the average range relative to monolingual English-speaking norms. Following Directions (RS = 15, ScS = 6, CI = 4–8) and Semantic Relationships (RS = 5, ScS = 6, CI = 4–8) were the most difficult subtests in both languages, and the results were classified as low or very low in English as well.

**Figure 3:**
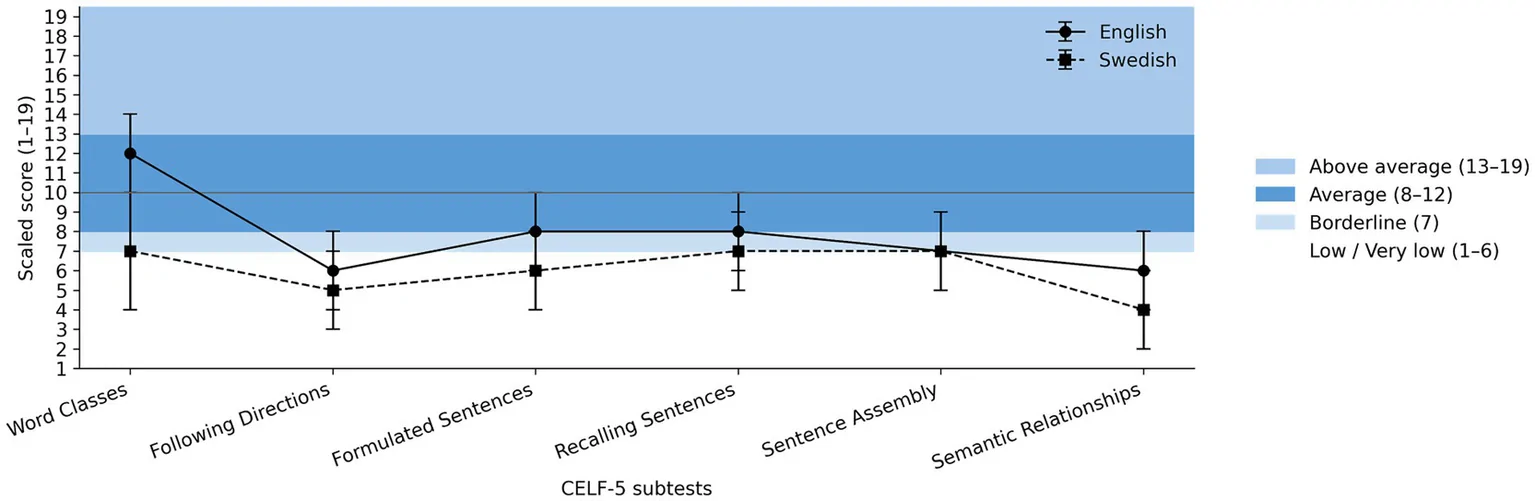
Stella’s results on the subtests on CELF-5 in English (solid line) and Swedish (dotted line). Scores are reported as Scaled Scores (mean = 10, SD = 3). The shaded bands represent CELF-5 normative categories.

### 4.4 Assessment of social responsiveness

The total *t*-score on the Social Responsiveness Scale was 63. This score falls within the mild range and indicates difficulties with reciprocal social behavior that may result in impairment of everyday social interaction. Based on the subscale scores, Stella appears to have the least difficulties with social awareness, *t*-score 57, and the most difficulties with social communication, *t*-score 64. The *t*-scores for the other subscales were 63 for social cognition, 60 for social motivation, and 62 for restricted interests and repetitive behavior.

### 4.5 Stella’s own thoughts about speaking English

During breaks between subtests and at the end of the test sessions, the SLPs and Stella spent time drawing and talking together. At the end of a session, when the test was administered in Swedish, the SLP explained to Stella that the researchers had also purchased the English version of the test and wanted to practice using it. The following excerpt is from Stella’s reaction to this.

Excerpt 1.

**Table:** 

1	Stella:	Jag vill vara “volunteer”*. (I want to volunteer.)
2	SLP:	För att göra uppgifterna? (To do the tasks?)
3	Stella:	Jo, för att jag är en “pro”* på att, på att prata engelska. (Yes, because I’m pro at, at speaking English.)

*Code-switching.

During a break in the English test session, the SLP asked Stella in which language she counts in her head. Stella answered that she counts in English and then spontaneously continued: “Så min minne e helt “English”, men jag talar svenska” (“So, my memory is completely English, but I speak Swedish”). The utterance contains a grammatical deviation (“min” instead of *mitt*), which would typically be expected to have been mastered at this age, and code-switching with the English word “English.” The next excerpt shows Stella’s reaction when the SLP tried to ask more about the topic. This part of the conversation was in English.

Excerpt 2.

**Table:** 

1	SLP:	If you have a memory, something that happened before, and you think about it, do you think about in Swedish or English?
2	Stella:	Sorry, I don’t have time. Zoe is more important. [the character she was drawing]
3	SLP:	Okay, I see.
4	Stella:	Sorry, but yeah, I just love Zoe. She’s literally my favorite character in the whole show.

In the following excerpt, the SLP asked Stella about speaking English after one test session. In the excerpt, Stella expresses her thoughts about speaking a foreign language. The conversation was in English.

Excerpt 3.

**Table:** 

1	SLP:	Do I remember correctly, or was it so that you spoke English and then you didn’t speak English for quite many years?
2	Stella:	Well…
3	SLP:	Or did you speak English to yourself, but you didn’t wanna speak it to anyone else?
4	Stella:	Well, I actually have no idea how I spoke English in the first place.
5	SLP:	Yeah. How you started?
6	Stella:	Yeah.
7	SLP:	Yeah, but you know, it’s not that uncommon. There are actually other kids who have done the same thing.
8	Stella:	No, I swear, this is like a disease or something.
9	SLP:	You think so?
10	Stella:	May… No, I don’t know. Diseases are usually with sickness, but maybe it could also be a…, but maybe it’s something called like English disease or something.
11	SLP:	But is it a good or bad thing to speak English when you are a kid?
12	Stella:	I have no idea. Maybe it… maybe it’s a good thing cause then you know more languages.
13	SLP:	That’s true.

## 5 Discussion

### 5.1 Screen language acquisition

The current case study describes Stella, a girl with ASD who appears to have unexpectedly acquired English through language input on screens. The area where Stella was growing up is entirely Swedish-speaking, and English is used neither in the family nor in the environment. The medical records are also consistent with the acquisition being screen-based. Therefore, based on our definition, we consider English to be a screen language for Stella. In Stella’s speech, there were clear prosodic features that originated from the screens, for example her accurate pronunciation in English, the rising pitch when she was counting in English, her resemblance to the cartoon character Peppa’s voice and her screen accent in Swedish. Stella’s language acquisition pattern, similar to the patterns described in previous case studies (Vulchanova et al., 2012; Kadiri and Anasse, 2023; Zhukova et al., 2023), can be interpreted as screen language acquisition.

### 5.2 Screen language dominance

Based on the interview, home videos, and medical records, the findings indicate that Stella preferred to speak English until she turned 5 years old. Several health professionals estimated that English was Stella’s stronger language between the ages of 2,5 and 5 years, during which time she did not speak Swedish on her own initiative. Moreover, her excellent English pronunciation and her tendency to pronounce Swedish names and words using English phonological patterns were considered striking and interpreted as indicating screen language dominance.

Between 3 and 5 years of age, Stella spoke exclusively in English. As standardized assessments could not be successfully administered, the relative strength of her two languages during this period remains uncertain, although observations suggested that English was the stronger language. A formal comparison was not possible until age nine, when assessment results showed stronger performance in English across all tested domains, suggesting screen language dominance. The fact that Stella has reached a higher level of proficiency in English suggests that she might not have reached her maximum linguistic capacity in Swedish, despite the support she has received. Stella’s own thoughts about her speaking English, e.g., counting in English and having an “English memory,” further suggest that she still has screen language dominance.

In conclusion, Stella’s language development aligns with our description of screen language dominance. When Stella was younger, she showed a clinically meaningful difference between her English and Swedish skills, as indicated by her language use and parental reports. At that time, screen language dominance could not be assessed formally because Stella did not cooperate with standardized testing. At age nine, however, the assessment results suggest that Stella still meets our criteria for screen language dominance, with the difference between English and Swedish CELF-5 Core Language Scores corresponding to more than one standard deviation, the threshold used to indicate meaningful dominance in this study. Two of the previously reported children (Kadiri and Anasse, 2023; Zhukova et al., 2023) also seem to align with our description, in that they showed clinically meaningful differences between their languages, although earlier studies did not use the term. On the other hand, one case (Vulchanova et al., 2012) showed equally strong proficiency in the screen language and the environmental language.

### 5.3 Screen accent

In addition to screen language acquisition, Stella has shown a screen accent. According to the mother, Stella watched children’s programs from Sweden and when she switched to speaking Swedish at age 5:0, she used an accent that is only used in Sweden and not in the Swedish-speaking parts of Finland. The home videos from when Stella was 5 to 7 years old, as well as the pediatric neurologist’s medical notes, appear to support the presence of a screen accent. Unlike screen language dominance, the screen accent seems to have vanished as Stella’s Swedish accent is at present more reminiscent of the accent of the environment. Vulchanova et al. (2012) also noted that the case they describe used a more formal variant of the local language that originated from the screen. Likewise, this is consistent with findings indicating that some children with ASD may have an unusually strong mastery of an accent or a linguistic variety acquired through screens (Kissine et al., 2019; Abd El-Raziq et al., 2024; Francis et al., 2024; Beccaria et al., 2025).

### 5.4 Possible factors influencing Stella’s screen language acquisition and language switch

Even though it is not possible to draw any general conclusions from Stella’s own thoughts regarding speaking English, her perspective provides new insights, as previous studies lack the children’s first-person perspectives on the matter. Stella’s reflections suggest that she does not know how she began speaking English. The importance of speaking Swedish has been highlighted by all health professionals, which may help explain her apparent hesitancy to discuss the topic, as suggested by occasions when she changed the topic of conversation. Moreover, Stella’s comment about having an “English disease” may reflect a perception that speaking English is undesirable. Although some of her comments may suggest pride in her English skills, the fact that her acquisition of English through screens has been viewed as a weakness rather than a strength due to screen language dominance may be reflected in her thoughts.

A unique aspect of Stella’s case is that she switched from only using the screen language to only using the environmental language. In addition to the uncertainty surrounding why Stella began speaking English, the cause of her language switch at age five is likewise unknown. One possible explanation is an increase in Swedish-language screen input, or that support from the environment began to yield results. Another possible reason is that, at the age of five, Stella may have developed the perception that speaking English was discouraged, as may be suggested by her subsequent refusal to speak English. Rather than representing a loss and later re-emergence of English proficiency, Stella’s language switch may reflect changes in language preference, communicative context, social adaptation, or language use.

Previous studies have proposed different factors influencing screen language acquisition, several of which align with Stella’s language development. Her language development may have been influenced by her special interests, which have been proposed as a contributing factor in previous research (Vulchanova et al., 2012; Kissine et al., 2019; Zhukova et al., 2023; Hindi and Meir, 2025). Stella’s mother believes that Stella felt that the English world on the screen was safer than her environment. Thus, an emotional event may have contributed to her preference for English, consistent with Hindi and Meir’s (2025) suggestion that an emotional event may influence language choice. Although Stella might enjoy interacting with others, it may still be easier for her to analyze and acquire language without interaction. Several previous studies have offered similar explanations regarding how the lack of social factors may facilitate language acquisition (Vulchanova et al., 2012; Kissine, 2021; Dumont et al., 2024; Francis et al., 2024; Gagnon et al., 2026; Mottron et al., 2026). Stella watched the same shows and episodes repeatedly, and consequently repeated content may represent an important contributing factor, as previous studies have also suggested (Kissine, 2021; Dumont et al., 2024; Francis et al., 2024; Gagnon et al., 2026). The interview and video material further suggest a strong ability to imitate prosodic features.

We suggest that delayed echolalia could be an additional contributing factor in Stella’s screen language acquisition. The description of Stella’s language development shows similarities to the account provided by Mottron et al. (2021), who describe how children with ASD may gradually modify reused utterances to produce contextually more appropriate responses. Given the methodological approach used in this study, it is difficult to determine the extent to which Stella’s speech consisted of delayed echolalia. Nevertheless, the data contain some examples suggesting the presence of delayed echolalia in her speech. The parent interview, the home videos, and the medical records all indicate that Stella acquired songs and rhymes from the screens. In addition, the mother’s observation about Stella using phrases resembling expressions commonly used in “Peppa Pig” suggests that delayed echolalia may have contributed to her early language development. The home videos contain an example of this. Although Stella’s early language production included delayed echolalia, the assessment of Stella’s English skills at age nine indicates that Stella’s language proficiency acquired through screens extends beyond simply reusing language from screens. Nevertheless, the proposed link between delayed echolalia and screen language acquisition remains speculative, as it is based on observations from a single case.

Although previous research has proposed possible factors influencing screen language acquisition, the precise mechanisms remain uncertain and require systematic investigation. Delayed echolalia, special interests, emotional safety, and reduced social demands should currently be regarded as tentative hypotheses. These factors appear to be linked to autistic traits, and thus an expanded understanding of language development in children with ASD would also contribute to a better understanding of screen language acquisition. Therefore, further research is needed.

### 5.5 Conceptual considerations and directions for further research

To study this topic further, it is crucial to establish clear terminology and definitions for the phenomenon, commonly referred to as unexpected bilingualism. Previous studies have primarily reported screen language acquisition in children with ASD, which has led to the phenomenon being associated with ASD. However, there is currently insufficient evidence to conclude that language acquisition through screens is unique to ASD. Evidence from Swedish preschools, together with anecdotal reports from Finland and Sweden, suggests that typically developing children may also use English in everyday communication (Larsson et al., 2023; Mustonen, 2024; Tenfält, 2024). Nevertheless, there may be differences in the screen language input children receive depending on the culture and linguistic context. Consequently, future research should investigate the phenomenon across different cultural contexts, diagnostic groups, linguistic backgrounds, communication profiles, screen formats, and levels of interpersonal language exposure.

If the term unexpected bilingualism refers mainly to learning a foreign language through screens, distinguishing between children with ASD and typically developing children becomes difficult. There is a lack of studies examining how proficient typically developing children can become in a foreign language through screens, making it challenging to determine what constitutes unexpectedly strong screen-based language skills. Given indications that screen language acquisition may also occur in typically developing children, it remains unclear why the phenomenon is often considered specifically associated with ASD. In our case, as well as in the case study by Zhukova et al. (2023), it is the level of the screen language and the relatively low level of the environmental language that appear to be particularly notable. We hypothesize that, in some children with ASD, language learning through interaction may be relatively slower than learning from screen-based input. This mechanism may contribute to the development of screen language dominance, as observed in Stella and in the case described by Zhukova et al. (2023).

We suggest distinguishing between screen language acquisition and screen language dominance. According to Treffers-Daller (2019), language dominance can be measured using either direct measures of relative language proficiency or indirect measures based on language exposure and use. Because children are unlikely to receive more input in the screen language than in the environmental language, direct assessment of relative language proficiency may be the most useful approach for identifying screen language dominance. However, when standardized assessments are not feasible, caregivers or professionals may suspect screen language dominance based on observations and the child’s language history. Further research and discussion are needed to evaluate and refine these proposed conceptual definitions.

Based on our findings, the use of delayed echolalia and unexpectedly good prosody and pronunciation, possibly at a level that is difficult to distinguish from typical first-language use, may represent candidate features warranting further research. Moreover, an important distinction may lie in the use of the screen language. Persistent use of a screen language in some children with ASD, regardless of the communicative context or the communication partner, may reflect underlying pragmatic difficulties, whereas typically developing children tend to use the screen language only in a specific context, such as during play. A related distinction between persistent and flexible use of a screen language concerns the impact of screen language acquisition on the children’s communicative participation. For typically developing children, as well as for children with ASD who can flexibly use both the screen language and the environmental language, the screen language skills may be considered a strength. In contrast, some children with screen language dominance may benefit from support to develop skills in the language used in their everyday environment. Children may, as Stella did, develop from showing screen language dominance and persistent use of the screen language to flexible use of both languages. It is possible that the severity of autism-related traits influences the extent to which communicative participation is affected by screen language dominance. Stella’s score within the mild range on the the Social Responsiveness Scale may have facilitated her adaptation to the environmental language by age five. Although Stella’s current language use suggests that communicative participation is no longer markedly affected, the assessment results do not allow conclusions regarding the presence of general functional English dominance or whether she would communicate more effectively in English than in Swedish.

The present case study, together with earlier studies on this topic, raises questions regarding the nature of screen language acquisition:

- What characterizes screen language acquisition in children with ASD?
- How prevalent is screen language acquisition among children with and without ASD?
- How prevalent is screen language dominance among children with and without ASD?

To help answer these questions, we suggest that future research:

- Address whether delayed echolalia or special interests may play a significant role in the child’s language development and the extent to which the child exhibits autistic traits.
- Include children with and without ASD.
- Describe the levels of both languages in order to identify potential screen language dominance and possible risk of reduced communicative participation.
- Acknowledge the need to differentiate between screen language acquisition, screen language dominance, and the use of a screen accent, a differentiation that has received limited attention in previous research.

### 5.6 Clinical implications

With clear conceptual definitions, future research may help to inform guidelines for SLPs working with children who show screen language acquisition or screen language dominance. The negative linguistic consequences of screen time are highlighted in the literature (Krcmar et al., 2007; Hill et al., 2020; Supanitayanon et al., 2020; Sundqvist et al., 2025), and concerns have also been raised regarding the connection between screen exposure and social skills (Alrahili et al., 2021; Chen et al., 2021). Consequently, children’s excessive screen time may contribute to feelings of failure among caregivers. Future research should aim to create guidelines regarding screen language input and screen exposure for children with ASD. At present, there is no consensus on whether screen exposure should be minimized or on the recommended language of screen input for children with ASD. As Hindi and Meir (2025) speculated on a possible bridge between acquiring a foreign language from screens and developing proficiency in the mother tongue, it remains possible that language input from screens in a foreign language may be beneficial for some children, although this has yet to be established empirically. Additionally, this raises questions regarding the choice of therapy language and whether the screen language should also be assessed when possible. Based on their findings, Hindi and Meir (2025) recommend assessing multilingual children in both their languages, regardless of whether the language was acquired from the environment or through screens. The present case suggests that assessment of screen language skills may be relevant when a child shows unexpected proficiency in a foreign language, even if the child refuses to speak it, because standard language assessments in the environmental language may underestimate overall linguistic capacity. Therefore, findings from the present case suggest that clinical attention may be directed toward communicative participation, environmental language access, family communication, and the child’s preferences, rather than solely toward whether a child’s language development conforms to expected bilingual trajectories.

Despite the highlighted negative linguistic consequences, our findings suggest that screen exposure may represent a meaningful source of language input for some children. Consequently, future research could explore whether screen-based approaches might be useful for children who appear to learn particularly well from screen-based language input, for example, by exploring the use of screens as a model for language learning. Nevertheless, a case study cannot establish the effectiveness or safety of screen-based interventions; therefore, this proposition requires further investigation through controlled and participatory studies.

### 5.7 Limitations and strengths

A limitation of this study is that some of the data rely on parental reports, which may include unconscious selection of materials. The mother selected which videos to share, and therefore, these may overrepresent moments where Stella used English or demonstrated advanced language, while underrepresenting struggles. The available video material was relatively sparse and cannot be assumed to reflect Stella’s everyday language use across contexts. Although the amount of available video material was limited, the possibility of selection bias should nevertheless be acknowledged. However, the medical records may partially mitigate this limitation as they are written by independent professionals without selection and support the developmental pattern observed in the home videos. In addition, insights from a single case cannot be generalized to groups of autistic children. This study does not allow any conclusion about how common screen language acquisition is among children with ASD or typically developing children; it serves only to generate hypotheses. Case studies can, however, enable a deeper understanding of a child’s language development and provide important insights for further research. Despite the three-month gap between test administrations, a potential order effect should be acknowledged. Furthermore, the use of two different examiners may have introduced examiner effects. The same subtests were administered in Swedish and English. Although these subtests are intended to assess comparable language domains across language versions, the Swedish and English editions are based on different linguistic adaptations and normative samples. Therefore, direct comparison between scores should be interpreted with caution. In particular, similar normative classifications across test editions do not necessarily imply equivalent underlying performance, as the normative bands are derived from different populations. Furthermore, the interpretation of the results is limited by the fact that the English version of the CELF-5 was normed on monolingual English-speaking children in North America, whereas Stella is not a native English speaker.

The fact that we have access to medical records from throughout Stella’s childhood from several different health professionals, as well as videos that support the mother’s description of Stella’s language development, strengthens the study’s findings and contributes to a better understanding of screen language dominance. Previous case studies lack more detailed information about children’s language development and a comprehensive description of their language use in different languages. In addition, they do not describe whether the children have received speech and language therapy, nor do they include any language assessments conducted prior to the study. Furthermore, the child’s own thoughts provide valuable insight into the phenomenon. A particular strength was the triangulation of findings across multiple data sources, which increased confidence in the interpretation of the results. Our efforts to problematize and clarify the terminology concerning screen language acquisition constitute a contribution to the field, and we invite others to continue examining and refining these conceptual definitions.

## 6 Conclusion

The findings from this case study indicate that Stella has acquired English as a screen language, previously used a screen accent in Swedish, and demonstrates screen language dominance. The language assessments unexpectedly indicate that Stella has a higher proficiency in the screen language compared to the language of the environment at age nine, despite receiving a range of support in Swedish. Our findings raise many questions about terminology, definitions and salient features regarding screen language acquisition and highlight the need for further research including children with and without ASD. Despite reported negative associations with screens, some children with ASD may be able to acquire language through screens, offering potential support for language development and emphasizing the need for further examination of the benefits and risks of screen language input and exposure. Screen language dominance may negatively affect the children’s communicative participation, and therefore, there is a need for evidence-based advice regarding how to best support the development of these children.

## Data Availability

The datasets presented in this article are not readily available because they include sensitive, potentially identifiable information. Requests to access the datasets should be directed to Emma Forsblom, emma.forsblom@abo.fi.
